# The Primary Prevention of Venous Thromboembolism in Patients with COVID-19 in Japan: Current Status and Future Perspective

**DOI:** 10.3400/avd.ra.20-00145

**Published:** 2021-03-25

**Authors:** Yugo Yamashita, Norikazu Yamada, Makoto Mo

**Affiliations:** 1 Department of Cardiovascular Medicine, Graduate School of Medicine, Kyoto University, Kyoto, Kyoto, Japan; 2 Department of Cardiology, Kuwana City Medical Center, Kuwana, Mie, Japan; 3 Department of Cardiovascular Surgery, Yokohama Minami Kyosai Hospital, Yokohama, Kanagawa, Japan

**Keywords:** COVID-19, venous thromboembolism, prevention, anticoagulation

## Abstract

Coronavirus disease 2019 (COVID-19) has become a huge threat worldwide as a pandemic, which could also cause venous thromboembolism (VTE), including pulmonary embolism (PE). On the basis of the concept of the high risk for VTE in patients with COVID-19, some studies reported the potential benefit of anticoagulation for the primary prevention of VTE. However, optimal strategies for the prevention of VTE in COVID-19 still remain unknown. Additionally, ethnic differences may have notable implications in the presentation of VTE. Very recently, in the Japanese Society of Phlebology and Japanese Society of Pulmonary Embolism Research, a questionnaire surveillance for COVID-19 and VTE was conducted, which revealed that the vast majority of the institutions did not have specific recommendations for the prevention of VTE with anticoagulation, the incidence rate of VTE was 0.6% (7/1243), and that of PE was 0.4% (5/1243). The current questionnaire surveillance has suggested that the management strategies for the prevention of VTE by anticoagulation in COVID-19 could widely vary according to institutions, and the number of patients diagnosed as VTE in COVID-19 in Japan was quite small compared with reports from other countries. Further studies, including cohort/registry-based studies, are warranted to confirm these results.

## COVID-19 and Venous Thromboembolism

Coronavirus disease 2019 (COVID-19) was first reported in Wuhan, China, in December 2019, which has become a huge threat worldwide as a pandemic.^1,2)^ COVID-19 is a viral illness caused by severe acute respiratory syndrome coronavirus 2, the main pathophysiology is a respiratory infectious disease, and COVID-19 could also cause cardiovascular complications.^3,4)^ Coagulopathy in patients with COVID-19 has been reported,^5)^ which could lead to thromboembolic complications.^6)^ In particular, several studies reported high prevalence of venous thromboembolism (VTE), including pulmonary embolism (PE), in hospitalized patients with COVID-19.^7–9)^ In addition, a recent autopsy study reported that more than half of the patients with COVID-19 had VTE, and some of them died because of PE.^10)^ Thus, patients with COVID-19 have been recognized as being at a high risk for VTE.

## Prevention of VTE in COVID-19

On the basis of the concept of the high risk for VTE in patients with COVID-19, the primary prevention of VTE in COVID-19, including anticoagulation, has been drawing attention. Some studies reported that the use of anticoagulation was associated with reduced mortality in hospitalized patients with COVID-19,^11,12)^ suggesting the potential benefit of anticoagulation for the prevention of thromboembolism in the management of COVID-19. In line with these reports, several consensus statements have recommended systematic pharmacological thromboprophylaxis in all patients who require hospital admission for COVID-19.^13,14)^ However, optimal strategies for the prevention of VTE in COVID-19 still remain unknown, which can be revealed through several ongoing randomized clinical trials in the near future (NCT04345848, NCT04344756, NCT04359277, NCT04362085, NCT04367831, NCT04377997, NCT04394377, and NCT04373707).

## The Risk of VTE and Bleeding with Anticoagulation in Asians

Historically, VTE has been considered a relatively uncommon disease among Asian populations. Some previous studies suggested lower incidences of VTE in Asian population compared with Caucasian population.^15–18)^ Ethnic differences, distinct resource availability, and treatment patterns may have notable implications in the presentation of VTE. In addition, there is a concern that anticoagulation might be associated with a higher risk of bleeding particularly in Asians than in Caucasians. In fact, previous studies have shown markedly higher rates of major bleeding in Asian patients than in non-Asian patients receiving anticoagulation for conditions such as atrial fibrillation.^19,20)^ Thus, it is important to take a good balance between the risk of thrombosis and bleeding with consideration of the ethnic difference.

## VTE in COVID-19 in Japan

Unfortunately, there is a scarcity of data on the current status of VTE in patients with COVID-19 in Japan. In the first place, the incidence of VTE in patients hospitalized for COVID-19 is completely unclear in Japan. Furthermore, although some institutions in Japan might have developed management strategies for anticoagulation in patients with COVID-19, the current management strategies in each institution have been unclear. These issues could be critically important to explore the establishment for optimal strategies for the prevention of VTE by anticoagulation in Japan.

Very recently, in a collaborative effort with the Japanese Society of Phlebology (JSP) and Japanese Society of Pulmonary Embolism Research (JaSPER), a questionnaire surveillance for COVID-19 and VTE was conducted, which revealed the current status of the management strategies for anticoagulation in each institution and estimated incidence of VTE in Japan. In July 2020, a questionnaire regarding management strategies for anticoagulation in each institution, the number of patients hospitalized for COVID-19, and the number of patients who developed VTE after diagnosis of COVID-19 from March 2020 to June 2020 was distributed among the members of JSP and JaSPER through e-mails. The data were collected from each institution using an electronic report form in a web-based database system. The current questionnaire surveillance was approved by the ethics committee of Kuwana City Medical Center (Approval number: 2020-168).

**Figure 1:**
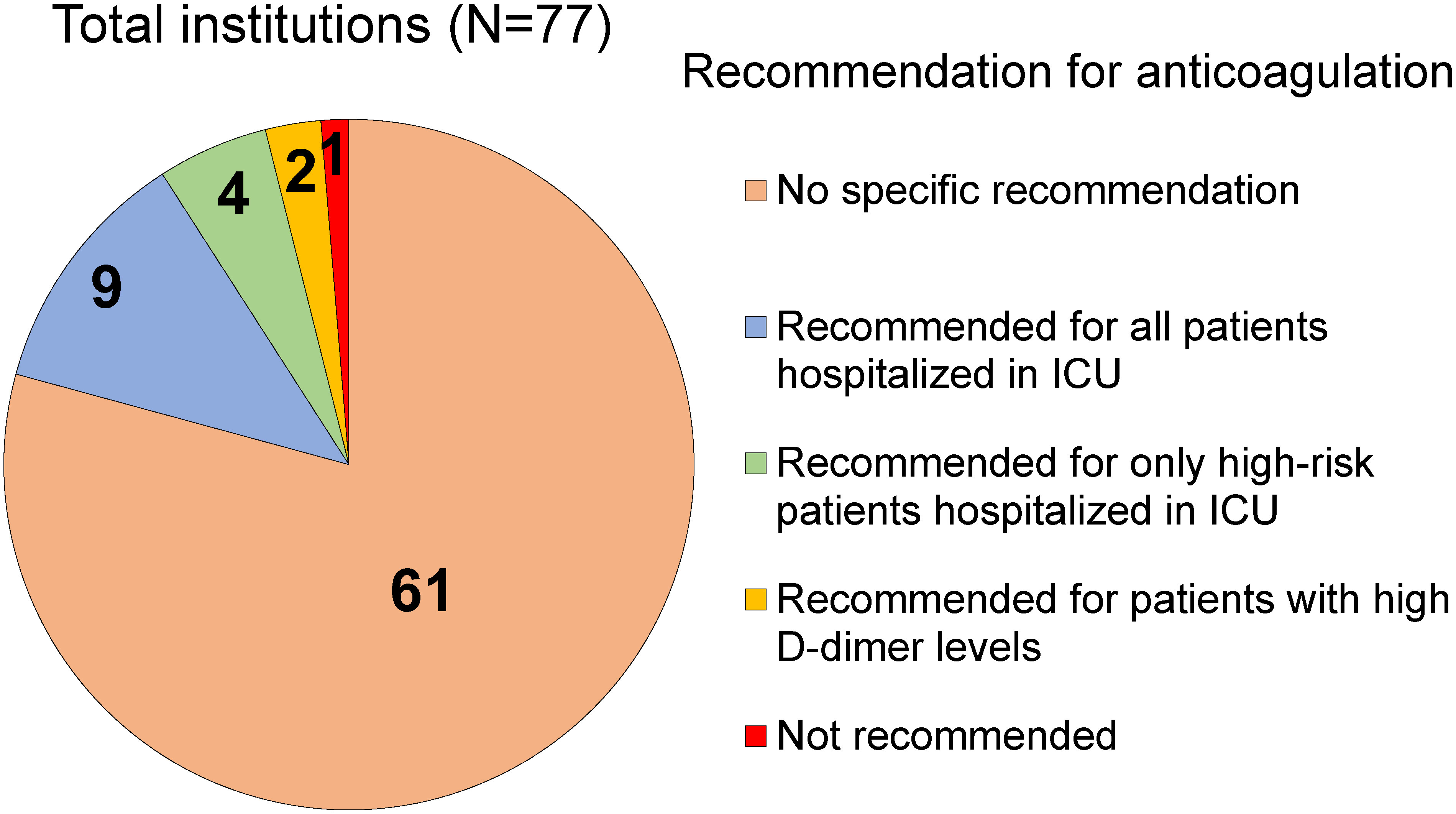
Fig. 1 The specific recommendation for the primary prevention of VTE by anticoagulation according to each institution.

**Figure 2:**
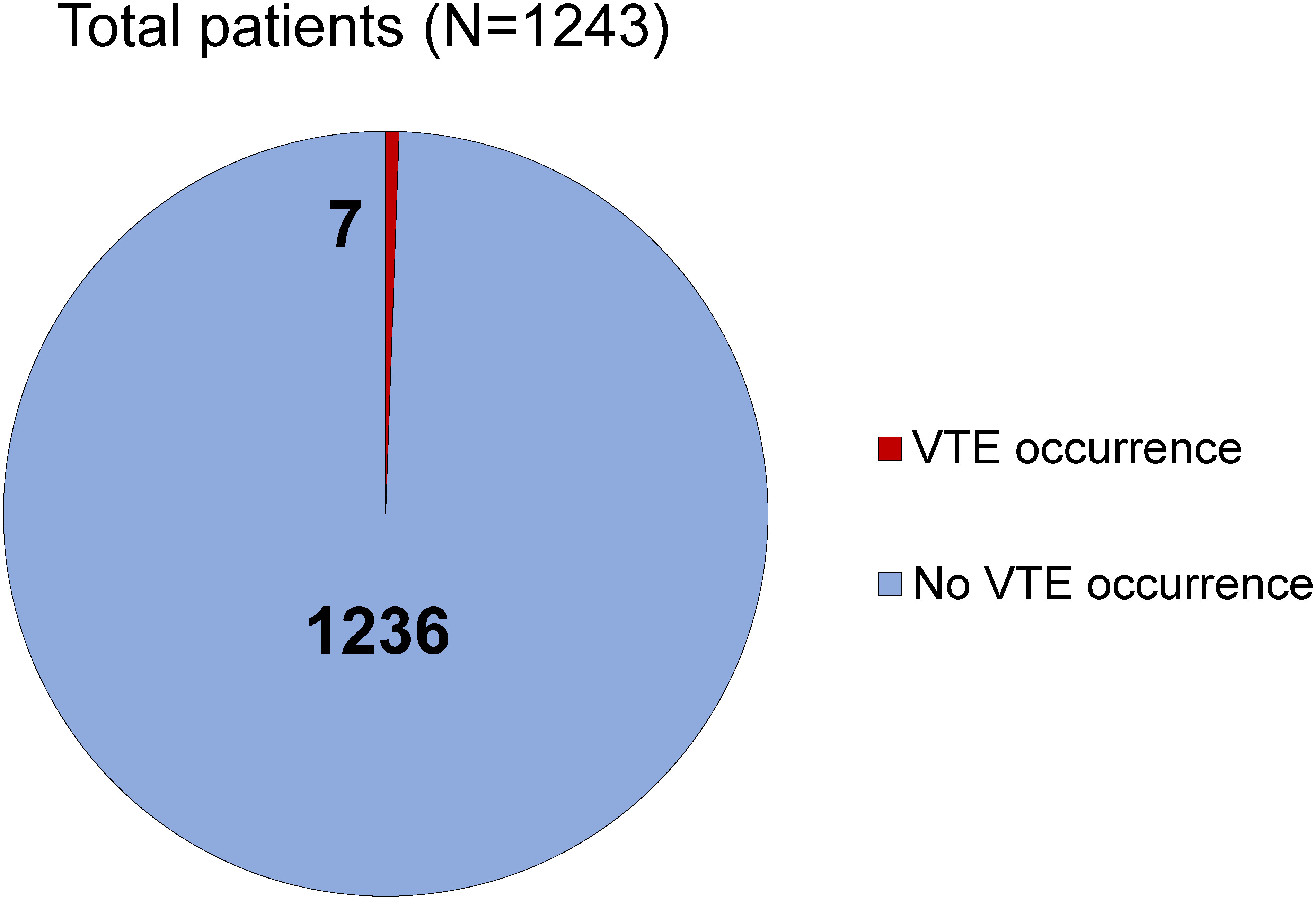
Fig. 2 Total number of VTE among all patients.

A total of 837 institutions were included, and the total response rate was 9.2% (77/837), which included 32% of university hospitals (25/77) and 42% of certified institutions for infectious diseases (32/77). Among 77 institutions, only 16 institutions had specific recommendations for the prevention of VTE with anticoagulation, and the rest of the institutions (79%) did not have specific recommendations (**Fig. 1**), which suggest that decision-making was left to the discretion of the doctors in charge. In the questionnaire surveillance, a total of 1243 patients with COVID-19 among 77 institutions were evaluated, and 7 patients had VTE, which consisted of 5 PE (**Fig. 2**). The incidence rate of VTE was 0.6% (7/1243), and that of PE was 0.4% (5/1243). Even if only patients with COVID-19 in certified institutions for infectious diseases (N=32) were evaluated, the incidence rate of VTE was 0.7% (5/713), and that of PE was 0.4% (3/713), which seemed to be almost consistent with the main results.

ICU: intensive care unit, VTE: venous thromboembolism

VTE: venous thromboembolism

The current questionnaire surveillance has suggested that management strategies for the prevention of VTE by anticoagulation in COVID-19 could wildly vary according to institutions, and the number of patients diagnosed as VTE in COVID-19 in Japan was quite small compared with reports from other countries. It has been unknown whether these results suggest the under-diagnosis of VTE in COVID-19 or actual lower prevalence of VTE in Japan. Furthermore, there might be many factors influencing the current results, including the severity of COVID-19 (target population) and the status of prevention by anticoagulation. Further studies, including cohort/registry-based studies, are warranted to confirm these results. Currently, a registry-based study focusing on patients assessed by imaging examinations is ongoing (UMIN000042235: Venous Thromboembolism in Patients with COVID-19 in Japan Study).

## Current Status of Anticoagulation Therapy in Japan

Another important issue is that low-molecular-weight heparin (LMWH) is recommended for the prevention and treatment of VTE in Western countries, although the use of LMWH is not covered by Japanese national insurance, except for specific conditions such as the primary prevention of VTE after surgery, and unfractionated heparin (UFH) is used in Japan. The advantages of LMWH over UFH include less injections and less heparin-induced thrombocytopenia. Thus, most patients in Western countries are given LMWH as the first choice. The clinical guidance on the diagnosis, prevention, and treatment of VTE in hospitalized patients with COVID-19 from the International Society on Thrombosis and Haemostasis states that a universal strategy of routine thromboprophylaxis with standard-dose UFH or LMWH should be used after careful assessment of bleeding risk, with LMWH as the preferred agent. Although the primary prevention of VTE by anticoagulation also seems to be recommended in Japan, the optimal types, and intensity of anticoagulants remain uncertain in daily clinical practice. Considering the uncertainty in VTE in COVID-19 and anticoagulation in Japan, further research is strongly and urgently needed in the near future to clarify the risk of VTE and optimal management strategies for the primary prevention of VTE.

## References

[1] Guan WJ, Ni ZY, Hu Y, et al. Clinical characteristics of coronavirus disease 2019 in China. N Engl J Med 2020; 382: 1708-20. 32109013 10.1056/NEJMoa2002032 PMC7092819

[2] Huang C, Wang Y, Li X, et al. Clinical features of patients infected with 2019 novel coronavirus in Wuhan, China. Lancet 2020; 395: 497-506. 31986264 10.1016/S0140-6736(20)30183-5 PMC7159299

[3] Clerkin KJ, Fried JA, Raikhelkar J, et al. COVID-19 and cardiovascular disease. Circulation 2020; 141: 1648-55. 32200663 10.1161/CIRCULATIONAHA.120.046941

[4] Driggin E, Madhavan MV, Bikdeli B, et al. Cardiovascular considerations for patients, health care workers, and health systems during the COVID-19 pandemic. J Am Coll Cardiol 2020; 75: 2352-71. 32201335 10.1016/j.jacc.2020.03.031 PMC7198856

[5] Zhang Y, Xiao M, Zhang S, et al. Coagulopathy and antiphospholipid antibodies in patients with Covid-19. N Engl J Med 2020; 382: e38. 32268022 10.1056/NEJMc2007575 PMC7161262

[6] Tang N, Li D, Wang X, et al. Abnormal coagulation parameters are associated with poor prognosis in patients with novel coronavirus pneumonia. J Thromb Haemost 2020; 18: 844-7. 32073213 10.1111/jth.14768 PMC7166509

[7] Cui S, Chen S, Li X, et al. Prevalence of venous thromboembolism in patients with severe novel coronavirus pneumonia. J Thromb Haemost 2020; 18: 1421-4. 32271988 10.1111/jth.14830 PMC7262324

[8] Klok FA, Kruip M, van der Meer NJM, et al. Confirmation of the high cumulative incidence of thrombotic complications in critically ill ICU patients with COVID-19: an updated analysis. Thromb Res 2020; 191: 148-50. 32381264 10.1016/j.thromres.2020.04.041 PMC7192101

[9] Llitjos JF, Leclerc M, Chochois C, et al. High incidence of venous thromboembolic events in anticoagulated severe COVID-19 patients. J Thromb Haemost 2020; 18: 1743-6. 32320517 10.1111/jth.14869 PMC7264774

[10] Wichmann D, Sperhake JP, Lutgehetmann M, et al. Autopsy findings and venous thromboembolism in patients with COVID-19: a prospective cohort study. Ann Intern Med 2020; 173: 268-77. 32374815 10.7326/M20-2003 PMC7240772

[11] Tang N, Bai H, Chen X, et al. Anticoagulant treatment is associated with decreased mortality in severe coronavirus disease 2019 patients with coagulopathy. J Thromb Haemost 2020; 18: 1094-9. 32220112 10.1111/jth.14817 PMC9906401

[12] Paranjpe I, Fuster V, Lala A, et al. Association of treatment dose anticoagulation with in-hospital survival among hospitalized patients with COVID-19. J Am Coll Cardiol 2020; 76: 122-4. 32387623 10.1016/j.jacc.2020.05.001 PMC7202841

[13] Spyropoulos AC, Levy JH, Ageno W, et al. Scientific and Standardization Committee communication: clinical guidance on the diagnosis, prevention, and treatment of venous thromboembolism in hospitalized patients with COVID-19. J Thromb Haemost 2020; 18: 1859-65. 32459046 10.1111/jth.14929 PMC7283841

[14] Zhai Z, Li C, Chen Y, et al. Prevention and treatment of venous thromboembolism associated with Coronavirus disease 2019 infection: a consensus statement before guidelines. Thromb Haemost 2020; 120: 937-48. 32316065 10.1055/s-0040-1710019 PMC7295267

[15] Liao S, Woulfe T, Hyder S, et al. Incidence of venous thromboembolism in different ethnic groups: a regional direct comparison study. J Thromb Haemost 2014; 12: 214-9. 24283769 10.1111/jth.12464

[16] White RH, Zhou H, Murin S, et al. Effect of ethnicity and gender on the incidence of venous thromboembolism in a diverse population in California in 1996. Thromb Haemost 2005; 93: 298-305. 15711746 10.1160/TH04-08-0506

[17] Stein PD, Kayali F, Olson RE, et al. Pulmonary thromboembolism in Asians/Pacific Islanders in the United States: analysis of data from the National Hospital Discharge Survey and the United States Bureau of the Census. Am J Med 2004; 116: 435-42. 15047032 10.1016/j.amjmed.2003.11.020

[18] Klatsky AL, Armstrong MA, Poggi J. Risk of pulmonary embolism and/or deep venous thrombosis in Asian-Americans. Am J Cardiol 2000; 85: 1334-7. 10831950 10.1016/s0002-9149(00)00766-9

[19] Chiang CE, Wang KL, Lip GY. Stroke prevention in atrial fibrillation: an Asian perspective. Thromb Haemost 2014; 111: 789-97. 24500243 10.1160/TH13-11-0948

[20] Oldgren J, Healey JS, Ezekowitz M, et al. Variations in cause and management of atrial fibrillation in a prospective registry of 15,400 emergency department patients in 46 countries: the RE-LY Atrial Fibrillation Registry. Circulation 2014; 129: 1568-76. 24463370 10.1161/CIRCULATIONAHA.113.005451

